# Single-Molecule Sequencing of the *Drosophila serrata* Genome

**DOI:** 10.1534/g3.116.037598

**Published:** 2017-01-30

**Authors:** Scott L. Allen, Emily K. Delaney, Artyom Kopp, Stephen F. Chenoweth

**Affiliations:** * School of Biological Sciences, The University of Queensland, St Lucia, Queensland 4072, Australia; † Department of Evolution and Ecology, University of California, Davis, California 95616

**Keywords:** *Drosophila*, *montium*, PacBio, Celera, long reads, genome assembly

## Abstract

Long-read sequencing technology promises to greatly enhance *de novo* assembly of genomes for nonmodel species. Although the error rates of long reads have been a stumbling block, sequencing at high coverage permits the self-correction of many errors. Here, we sequence and *de novo* assemble the genome of *Drosophila serrata*, a species from the *montium* subgroup that has been well-studied for latitudinal clines, sexual selection, and gene expression, but which lacks a reference genome. Using 11 PacBio single-molecule real-time (SMRT cells), we generated 12 Gbp of raw sequence data comprising ∼65 × whole-genome coverage. Read lengths averaged 8940 bp (NRead50 12,200) with the longest read at 53 kbp. We self-corrected reads using the PBDagCon algorithm and assembled the genome using the MHAP algorithm within the PBcR assembler. Total genome length was 198 Mbp with an N50 just under 1 Mbp. Contigs displayed a high degree of chromosome arm-level conservation with the *D. melanogaster* genome and many could be sensibly placed on the *D. serrata* physical map. We also provide an initial annotation for this genome using *in silico* gene predictions that were supported by RNA-seq data.

Second-generation sequencing (2GS) platforms, such as Illumina sequencing-by-synthesis, have dramatically reduced genome sequencing costs while increasing throughput exponentially (Shendure and Ji 2008). The relatively low cost and massive throughput of 2GS platforms have paved the way for sequencing and *de novo* assembly of thousands of species’ genomes (Alkan *et al.* 2011). 2GS methods generate short reads (less than a few hundred base pairs in length) that have limitations for *de novo* genome assembly, where assembly is performed without the aid of a reference genome (Green 1997; Miller *et al.* 2008; Nagarajan and Pop 2013; Alkan *et al.* 2011). With short reads, *de novo* assembly is an inherently difficult computational problem because repetitive DNA sequences are often much longer than the length of each read (Ukkonen 1992). For instance, it has been estimated that short-read *de novo* assemblies could be missing up to 20% of sequence information because repeat DNA sequences can increase the number of misassembled and fragmented regions (Schatz *et al.* 2010; Alkan *et al.* 2011; Ukkonen 1992). One way to alleviate the problem of repetitive DNA in the *de novo* assembly process has been to incorporate a second set of mate-pair libraries with very long inserts (> 2 kbp) (Li *et al.* 2010; Chaisson *et al.* 2009; Simpson *et al.* 2009; Alkan *et al.* 2011; Butler *et al.* 2008). Mate-pair libraries can resolve repeats (Treangen and Salzberg 2012; Wetzel *et al.* 2011) and improve scaffolding (van Heesch *et al.* 2013), but paired-end contamination and insert size misestimation can also lead to misassemblies (Phillippy *et al.* 2008; Sahlin *et al.* 2016).

More recently, third-generation (3GS) single-molecule sequencing technologies, such as Pacific Biosciences’ (PacBio) SMRT sequencing and Oxford Nanopore’s MinION sequencing, which currently produce much longer reads of up to 54 kbp (Lee *et al.* 2014) and > 10 kbp (Quick *et al.* 2014), respectively, can overcome some of the shortcomings of 2GS *de novo* assembly (Berlin *et al.* 2015). Although long-read sequencing technology produces reads with a high error rate, ranging from 82.1% (Chin *et al.* 2011) to 84.6% accuracy (Rasko *et al.* 2011), sequencing errors occur at more or less random positions across long reads (Chin *et al.* 2013) and can be corrected with 2GS short-read data (Koren *et al.* 2012) or by using excess 3GS reads for self-correction (Chin *et al.* 2013).

In this paper, we use PacBio long-read sequencing to *de novo* assemble the genome of the fly, *Drosophila serrata*, which has been particularly well-studied from an evolutionary standpoint. *D. serrata* is a member of the *D. montium* subgroup, which split from the *D. melanogaster* subgroup ∼40 MYA (Tamura *et al.* 2004), and consists of an estimated 98 species (Brake and Bächli 2008). At present, only one draft genome assembly (*D. kikkawai*) is available (Chen *et al.* 2014) from this species-rich subgroup. *D. serrata* has a broad geographical distribution, ranging from Papua New Guinea to south eastern Australia and has emerged as a powerful model for addressing evolutionary questions such as the evolution of species borders (Blows and Hoffman 1993; Hallas *et al.* 2002; Magiafoglou *et al.* 2002) and climate adaptation (Frentiu and Chenoweth 2010; Latimer *et al.* 2011; Kellermann *et al.* 2009). The species has also been used to investigate sexual selection (Hine *et al.* 2002; Gosden and Chenoweth 2011; Frentiu and Chenoweth 2008; Chenoweth *et al.* 2015), male mate choice (Chenoweth and Blows 2003; Chenoweth *et al.* 2007), mate recognition (Higgie *et al.* 2000), sexual dimorphism (Chenoweth *et al.* 2008; Yassin *et al.* 2016), sexual conflict (Delcourt *et al.* 2009), and indirect genetic effects (Chenoweth *et al.* 2010b). In addtion, its cuticular hydrocarbons, which serve as contact pheromones (Chung *et al.* 2014), have been extensively used to develop novel multivariate quantitative genetic approaches for exploring genetic constraints on adaptation (Blows *et al.* 2004; Chenoweth *et al.* 2010a; McGuigan *et al.* 2011b; Rundle *et al.* 2009).

Despite the importance of *D. serrata* as a model for evolutionary research, our poor understanding of its genome remains a significant limitation. Linkage and physical genome maps are available (Stocker *et al.* 2012) and an expressed sequence tag (EST) library has been developed (Frentiu *et al.* 2009), but the species lacks a draft genome. Here, we report the sequencing and assembly of the *D. serrata* genome using exclusively PacBio SMRT technology. We also provide an initial annotation of the genome based on *in silco* gene predictors supported by empirical RNA-seq data. Our *de novo* genome and its annotation will provide a resource for ongoing population genomic and trait mapping studies in this species as well as facilitate broader studies of genome evolution in the family Drosophilidae.

## Materials and Methods

### Fly strains and DNA extraction

We sequenced a mix of ∼100 mg of males and females from a single inbred line that originated from Forster, Australia, and had been inbred via full-sib mating for 10 generations before being maintained at a large population size (*N* ∼ 250 individuals) (McGuigan *et al.* 2011b). A single further generation of full-sib inbreeding was applied before extraction of DNA. This same inbred line was used for the *D. serrata* linkage map, was the founding line for previous mutation accumulation studies (Latimer *et al.* 2015; McGuigan *et al.* 2011a, 2014a,b), and is fixed for the light female abdominal pigmentation phenotype mapped by Yassin *et al.* (2016). High molecular weight DNA was extracted from fly bodies (heads were excluded to reduce eye pigment contamination) using a QIAGEN Gentra Puregene Tissue Kit (Cat #158667), which produced fragments > 100 kbp (measured using pulsed-field gel electrophoresis). Two phenol–chloroform extractions were performed at the University of California, Davis at the DNA Technologies Core prior to preparation of a standard sequencing library.

### Genome sequencing and assembly

DNA was sequenced using 11 SMRT cells and P6-C4 chemistry on the PacBio RS II platform. In total, this produced ∼13 Gbp spanning 136,119 filtered subreads with a mean read length of 8840 bp and an N50 of 12,220 bp (Supplemental Material, Figure S1). The PacBio genome was assembled using the PBcR pipeline, which implements the MHAP algorithm within the Celera Assembler (version 8.3rc2) (Berlin *et al.* 2015), and polished with Quiver (GenomicConsensus version 0.9.2 and ConsensusCore version: 0.8.8) (Chin *et al.* 2013) in three steps: (1) errors were corrected in reads using PBDagCon, which requires at least 50 × genome coverage and utilizes the consensus of oversampled sequences (Chin *et al.* 2013); (2) overlapping sequences were assembled using MHAP and the Celera Assembler (Berlin *et al.* 2015); and (3) contigs were polished with Quiver to correct for spurious SNP calls and small indels (Chin *et al.* 2013). The “sensitive” setting was used for both read correction and genome assembly (Berlin *et al.* 2015) whereas the default settings were used for polishing with Quiver (Chin *et al.* 2013). We elected to correct all reads as opposed to the default longest 40 ×. The longest 25 × corrected reads were subsequently used for genome assembly. The PBDagCon correction was performed on a computer with 60 CPU cores and 1 TB of RAM; 58 CPU cores were used for the assembly and the amount of RAM used, although not tracked, was far less than machine capacity. Error correction with PBDagCon took ∼26 days. Assembly of corrected reads using MHAP and the Celera Assembler took ∼19 hr using 28 CPU cores. Our initial runs using the much faster error correction algorithm (HGAP) produced a slightly shorter assembly (194 Mbp compared to 198 Mbp) with a slightly lower N50 (0.88 Mbp *vs.* 0.95 Mbp). Therefore, we chose to use the more sensitive PBDagCon correction method.

### Transcriptome sequencing and assembly

The same inbred fly strain that was used for DNA sequencing was also used for adult mRNA sequencing to annotate the *D. serrata* genome. Adult males and females were transferred to fresh vials shortly after eclosion and held in groups of ∼25 where they were allowed to mate and lay eggs for 2 d. They were then sexed under light CO_2_ anesthesia and snap frozen using liquid nitrogen in groups of 10; at the time of freezing, all flies were assumed to be nonvirgins. Total RNA was extracted from each pool of flies using the standard TRIzol protocol. Initial quality assessment of the total RNA using a NanoDrop and gel electrophoresis indicated that the RNA was of high quality, this was later confirmed with a RNA integrity number > 7 (measured uisng a BioAnalyzer). RNA was stored at −80° for several days before being shipped for sequencing.

One male and one female 75 bp paired-end sequencing library was prepared using the TruSeq Stranded mRNA Library prep kit and sequenced on an Illumina NextSeq500 at the Ramaciotti Centre for Genomics, University of New South Wales, Australia. In total, 79 and 88 million reads were produced for males and females, respectively. Quality assessment of the RNA-seq data using FastQC (Andrews 2010) indicated that the reads were of a high quality and therefore no trimming of reads was performed. The transcriptome was *de novo* assembled for each sex separately using Trinity version 2.1.1 (Grabherr *et al.* 2011), where all reads were used and the jaccard_clip option was enabled to minimize gene fusion events caused by UTR overlap in high gene density regions.

### Annotation

Maker version 2.31.8 (Campbell *et al.* 2014; Holt and Yandell 2011) was used to annotate the PacBio genome via incorporation of *in silico* gene models detected by Augustus (Stanke and Morgenstern 2005) and/or SNAP (Johnson *et al.* 2008), the *de novo D. serrata* male and female transcriptomes, and protein sequences from 12 *Drosophila* species genomes (*D. ananassae* r1.04, *D. erecta* r1.04, *D. grimshawi* r1.3, *D. melanogaster* r6.07, *D. mojavensis* r1.04, *D. persmillis* r1.3, *D. pseudoobscura pseudoobscura* r3.03, *D. sechellia* 1.3, *D. simulans*, r2.01, *D. virilis* r1.03, *D. willistoni* r1.04, and *D. yakuba* r1.04) obtained from FlyBase (McQuilton *et al.* 2012; Attrill *et al.* 2016). Repeat masking was performed based on *D. melanogaster* training (Smit *et al.* 1996). Maker was run with default settings apart from allowing Maker to take extra steps to identify alternate splice variants and correct for erroneous gene fusion events.

### Data availability

All sequence data including PacBio and RNA-seq reads have been submitted to public repositories and are available via the *D. serrata* genome NCBI project accession PRJNA355616. The genome assembly and annotation tracks are available from http://www.chenowethlab.org. We also supply a list of *D. melanogaster* orthologs in Table S1. 

## Results and Discussion

To assemble a draft *D. serrata* genome, we sequenced DNA from a pool of adult males and females (that originated from a single inbred line) to a coverage of ∼65 × using PacBio long-read, SMRT sequencing technology. This produced 136,119 filtered subreads with a mean read length of 8940 bp and a read N50 of 12,200 bp that spanned > ∼13 Gbp (Figure S1). The PacBio reads were assembled using the MHAP algorithm within the Celera Assembler (Miller *et al.* 2008; Berlin *et al.* 2015) after self-correction using PBDagCon (Chin *et al.* 2013). The final genome was polished with a single iteration of Quiver (Chin *et al.* 2013) and consisted of 1360 contigs spanning > 198 Mbp with a GC content of 39.13% (Table 1). The longest contig was ∼7.3 Mbp and the N50 of all contigs was ∼0.95 Mbp. Flow cytometry studies suggest that species of the *montium* subgroup commonly have genome lengths over 200 Mbp (Gregory and Johnston 2008) with the estimate for the female *D. serrata* genome being ∼215 Mbp (0.22 pg). This estimate is in broad agreement with our assembly length of 198 Mbp for the female genome.

**Table 1 D.** *serrata* genome assembly statistics

Description	Statistic
Number of contigs	1360
Genome size (bp)	198,298,763
Longest contig (bp)	7,300,740
< 1 kbp	0.0%
1–10 kbp	3.3%
10–100 kbp	78.8%
100–1000 kbp	15.3%
> 1 Mbp	2.6%
N50 (bp)	942,627
GC content	39.13%

Contig length percentages refer to percent total length in each size bin.

### Completeness

Genome completeness was assessed using BUSCO gene set analysis version 2.0, which includes a set of 2799 genes specific to Diptera (Simao *et al.* 2015). The *D. serrata* assembly contained 96.2% of the BUSCO genes with 94.1% being complete single-copy (defined as complete when the gene’s length is within 2 SDs of the BUSCO group’s mean length) and 2.5% detected as fragmented. Only 1.3% of the BUSCO genes were not found in the *D. serrata* assembly (Table 2). Completeness of the *D. serrata* genome was similar to the reference *D. melanogaster* genome (version r6.05), which contained 98.7% complete BUSCO genes. As a further point of comparison, we computed BUSCO metrics for a recent PacBio-only assembly of the *D. melanogaster* ISO1 strain genome using all 790 contigs rather than the 132 that were constructed from > 50 reads only [http://www.cbcb.umd.edu/software/PBcR/MHAP/ (quivered full assembly)], and we also analyzed the only other member of the montium subgroup with a publicly available genome assembly, *D. kikkawai*, (https://www.hgsc.bcm.edu/arthropods/drosophila-modencode-project; NCBI PRJNA62319). Although these assemblies tended to contain marginally lower numbers of missing BUSCOs, metrics were generally very similar (Table 2), indicating a high level of completeness for the *D. serrata* assembly.

**Table 2:** BUSCO gene content assessment for *D. serrata* and two different *D. melanogaster* assemblies, version r6.05 from www.flybase.org, and the full ISO 1 PacBio assembly of Berlin *et al.* (2015) consisting of 790 contigs, also constructed with the PBcR pipeline

Category	*D. serrata*	*D. kikkawai*	*D. melanogaster*	
			r6.05	PacBio
Complete Single-copy BUSCOs (%)	94.1	97.1	98.2	97.7
Duplicated (%)	2.1	1.0	0.5	0.6
Fragmented BUSCOs (%)	2.5	1.2	0.8	0.8
Missing BUSCOs (%)	1.3	0.8	0.5	0.9

A total of 2799 BUSCOs were searched that form a set of highly conserved Dipteran genes. PacBio, Pacific Biosciences; BUSCO, Benchmarking Universal Single-Copy Ortholog.

### Fragmentation and misassemblies

Although our assembly consisted of 1360 contigs with a N50 of 0.94 Mbp, which was an N50 at the upper end of what might be expected for a short-read assembly, it is much lower than a recent PacBio-only assembly of the *D. melanogaster* genome (Berlin *et al.* 2015). There are several reasons why this might be the case. First, we report metrics on all contigs in the assembly rather than excluding those that incorporated < 50 reads, as was the case for the *D. melanogaster* assembly (Berlin *et al.* 2015) (132 contigs with an N50 of 13.6 M). Excluding such contigs resulted in a *D. serrata* assembly of only 273 contigs with a total genome length of 175 Mbp (*vs.* 198 Mbp) and an N50 of 1.4 Mbp. In this reduced assembly, half of the genome was represented in only 25 contigs, which is closer to the performance seen for *D. melanogaster*. While contigs with < 50 read support were generally short (median 23.5 kbp and range 6.3–110 kbp) and could be excluded in some cases on the basis of quality, when we examined the *D. serrata* annotation data, we saw that many of these contigs contained predicted genes that had RNA-seq support, including 14 complete single-copy BUSCOs. Therefore, we have retained all contigs in our assembly.

Second, although our N50 filtered subread length of 12,200 kbp is on a par with the *D. melanogaster* P5-C3 filtered subread lengths (12.2–14.2 kbp) (Kim *et al.* 2014), we had approximately half the coverage of the *D. melanogaster* assembly (65 × *vs.* 130 ×), which may have reduced our ability to span repetitive regions of the *D. serrata* genome. To examine this further, we reran the PBcR pipeline with *D. melanogaster* data from Kim *et al.* (2014) but downsampled it to 65 ×. We did not see genome contiguity drop to the levels seen for *D. serrata* (data not shown) and note that similar findings were observed by Chakraborty *et al.* (2016) (see their Figure 5). Therefore, it seems likely that the *D. serrata* genome, which is longer than that of *D. melanogaster*, may also be more complex due to longer repetitive regions. Therefore, adequate repeat-spanning coverage would presumably require additional very long reads to achieve the same assembly contiguity seen for *D. melanogaster*. A third factor possibly contributing to a higher degree of fragmentation in our assembly is residual heterozygosity, which may have been higher in our *D. serrata* line than the ISO1 *D. melanogaster* line.

**Figure 1:**
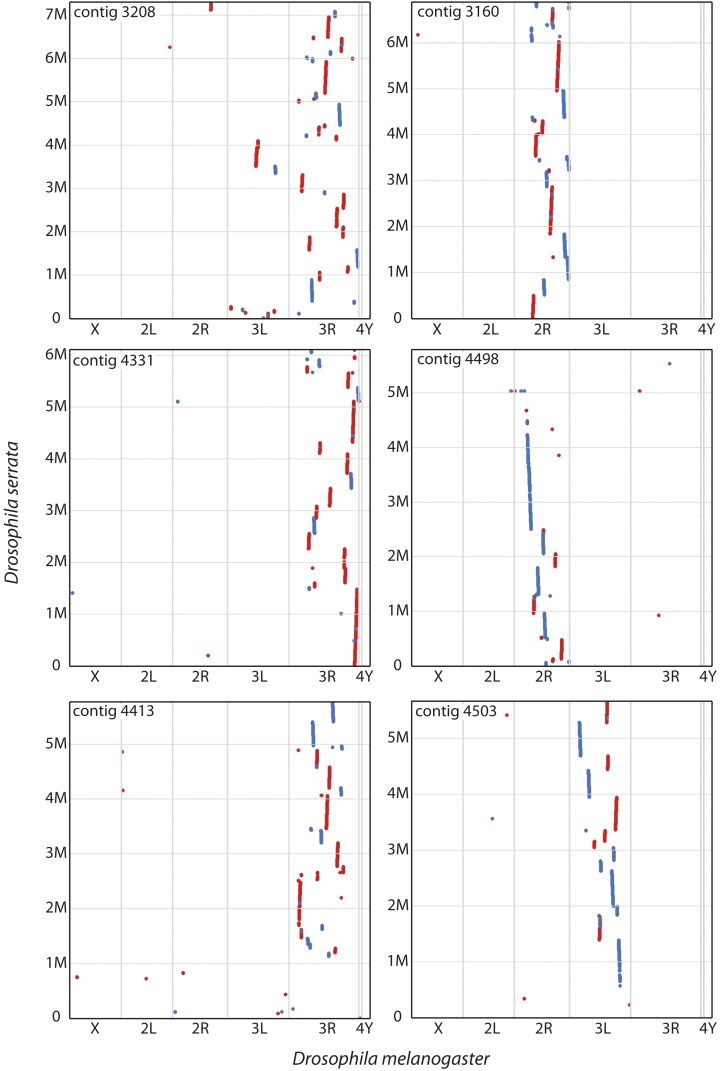
Alignment of the six longest contigs from the *D. serrata* assembly to *D. melanogaster* genome version 6.05. Red dots indicate a MUMmer alignment that matches to the *D. melanogaster* genome in the forward orientation; blue dots indicate a MUMmer alignment that matches to the *D. melanogaster* genome in the reverse orientation. M, million.

We used several methods to assess the quality of the genome with regards to misassemblies. First, because the *D. serrata* physical map indicates very strong chromosome arm-level conservation of gene content between *D. serrata* and *D. melanogaster* (Stocker *et al.* 2012), we examined possible misassemblies between chromosomal arms by aligning the six largest contigs (total length ∼37 Mbp) to the *D. melanogaster* genome using MUMmer (Kurtz *et al.* 2004). If there were no chromosome arm misplacements, then it was expected that each contig would align to a single *D. melanogaster* chromosome arm, albeit fragmented due to changes in gene order. This was largely the case (Figure 1), where each contig aligned to a single *D. melanogaster* chromosome arm but with minor sections of alignment to other chromosome arms toward the contig edges where repetitive elements were more likely to be found. The one major exception to this general pattern of conservation was found in the longest contig in the assembly, contig 3208, which aligned mainly to *D. melanogaster* 3R but contained an ∼600 kbp segment that aligned to *D. melanogaster* 3L. To test whether this was likely to be a misassembly, we searched the contig for previously published SNP markers that have been placed on the *D. serrata* linkage map. The marker m25 (Stocker *et al.* 2012), which maps to 3L, was located in the suspected misassembled region (contig 3208 and position 3,537,591) indicating that a misassembly rather than a genomic translocation rearrangement between 3R and 3L was most likely.

**Figure 2:**
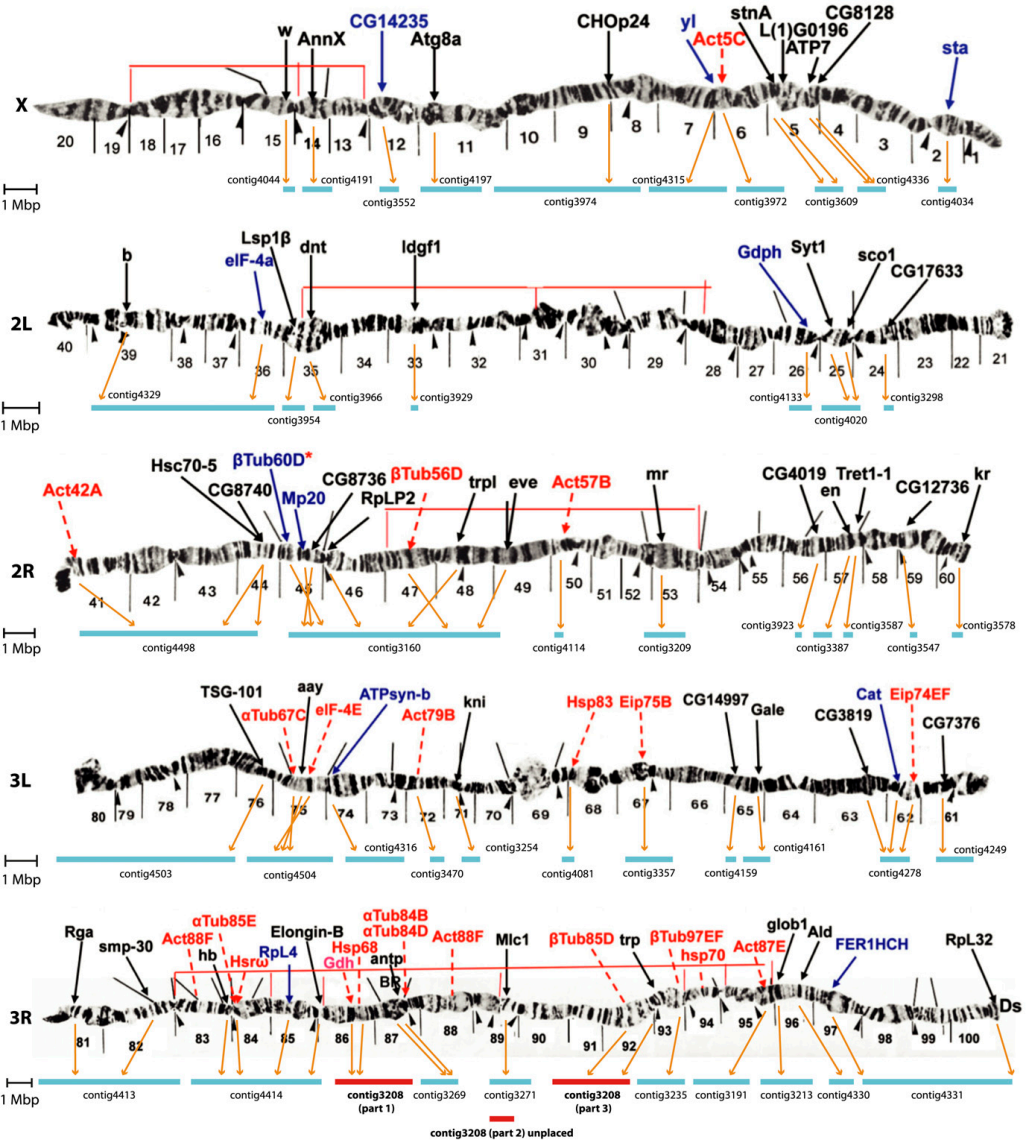
Comparison between the draft genome assembly and the physical *D. serrata* genome map, image is adapted from Stocker *et al.* (2012). Genes in red were mapped by Drosopoulou and Scouras (1995, 1998), Drosopoulou *et al.* (1996, 1997, 2002), and Pardali *et al.* (1996). Genes in blue are also included in the linkage map produced by Stocker *et al.* (2012). Thin red lines are inversions found by Stocker *et al.* (2004) and thin black lines are inversions found by Mavragani-Tsipidou *et al.* (1990). Contig3208 (shown in red), was split into three parts based on the misassembly; parts 1 and 3 aligned with *D. melanogaster* 3R and part 2 aligned with 3L (Figure 1). Markers Act88F and hsp70 were not mapped to contigs because the former appears twice and nomenclature changes meant we could not be certain exactly which gene hsp70 was referring to.

To further examine assembly quality, we compared our assembly to the entire physical genome map of *D. serrata* (Stocker *et al.* 2012), where *in situ* hybridization was used to physically locate 78 genes. We were able to assess possible misassemblies when a contig contained multiple physically mapped genes (11 contigs ranging in size from ∼1 to ∼6 Mbp). Using this approach, we observed no apparent chromosome arm-level assignment errors beyond that seen for contig 3208 (Figure 2). Furthermore, when contigs contained three or more physically mapped genes, gene order could be examined. We saw three cases of apparent gene order reversal (two on 2R and one on 3L). Interestingly, two of these regions map to the positions of known chromosomal inversions (Mavragani-Tsipidou *et al.* 1990), which is perhaps not unexpected given that different inbred lines were used for the physical map and genome sequencing. After considering these probable inversions, gene order and location appears to be largely correct for these 11 contigs at least. For contig 3208, each section that aligned to 3R could be placed on the physical map only after splitting the contig into three pieces based on the previously identified misassembly.

**Figure 3:**
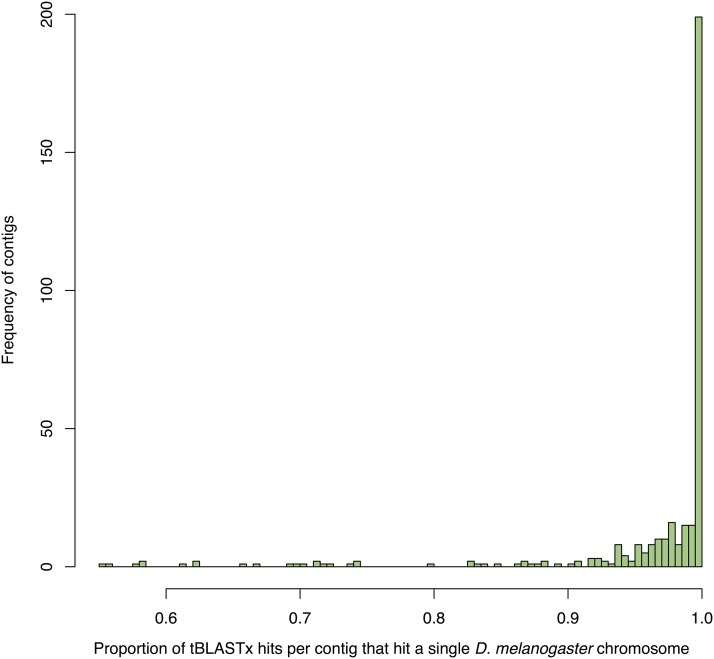
Comparison of *D. serrata* gene locations relative to *D. melanogaster*. On average, > 95% of tBLASTx hits to *D. melanogaster* genes (version 6.05) in each contig map to a single *D. melanogaster* arm.

The conservation of chromosome arm-level gene content was a common feature of the remaining contigs as well. For example, while only 354 contigs contained significant tBLASTx hits to at least one *D. melanogaster* gene (genome version 6.05), these contigs spanned 167 Mbp, and the vast majority had > 90% tBLASTx hits to a single *D. melanogaster* chromosomal arm (mean = 96.35% and median = 100%) (Figure 3). Furthermore, only 34 contigs displayed < 90% similarity to *D. melanogaster* and a linear regression where contig size predicted percent similarity indicated no significant relationship (F_(1,32)_ = 0.4003, *P* = 0.5314), suggesting that very large contigs were no more likely to be misassembled than short contigs.

### Annotation

To facilitate annotation of the *D. serrata* genome, we sequenced mRNA from male and female adult flies. The *in silico* gene predictors SNAP (Johnson *et al.* 2008) and Augustus (Stanke and Morgenstern 2005) found 22,718 and 15,984 genes, respectively. Of these *in silico* predicted genes, a total of 14,271 protein coding genes were sufficiently supported by RNA-seq and/or protein sequence data to be annotated by Maker2 (Holt and Yandell 2011). Maker scores annotations using the annotation edit distance (AED), a zero-to-one score where a value of zero indicates that the *in silico* annotation and the empirical evidence are in perfect agreement and a value of one indicates that the *in silico* annotation has no support from empirical data (Eilbeck *et al.* 2009). The AED for the *D. serrata* genome had a mean score of 0.18 and median of 0.13, suggesting that most annotations were of high quality with strong empirical support. While the number of genes we annotated in *D. serrata* is similar to the 13,929 protein coding genes that have currently been annotated in *D. melanogaster* (genome version 6.05), we annotated far fewer total transcripts (31,482 identified in *D. melanogaster* versus 16,202 in *D. serrata*) (Attrill *et al.* 2016); this is likely due to the larger number of tissue types and life stages for which *D. melanogaster* gene expression has been characterized with RNA-seq. For instance, considering that in *Drosophila* appreciable numbers of genes peak in expression during early life stages such as embryogenesis (Arbeitman *et al.* 2002), our use of adult fly RNA-seq data may mean that some such genes are yet to be annotated. Furthermore, as we used mRNA-seq, we have not yet annotated noncoding genes of which there are 3503 in the *D. melanogaster* genome (Attrill *et al.* 2016). Future RNA-seq datasets will be used to update the existing gene models.

We observed differences in gene, exon, and intron lengths between *D. serrata* and *D. melanogaster*. In *D. serrata*, there were on average 3.9 exons per protein coding gene and the gene, exon, and intron lengths were 4655, 451, and 699 bp respectively. Apart from average exon number, which does not differ between the two species, these values are lower than those for *D. melanogaster* protein coding genes (genome version 6.05), where the mean gene, exon, and intron lengths are 6962, 539, and 1704 bp, respectively (Attrill *et al.* 2016). The lower average intron length observed in *D. serrata* may be a consequence of annotating far fewer alternate splice variants. In total, coding sequence comprised 33.6% of the genome when including introns and 15.4% of the genome when considering only exons. Lower percentage intron content has been associated with overall longer genomes in Drosophilidae (Gregory and Johnston 2008), which is consistent with our observations here.

Many of the annotated genes in *D. serrata* were found to be putative orthologs of *D. melanogaster genes* (Table S1). In total, 10,995 (77%) were found to be orthologs via best reciprocal BLAST (Huynen and Bork 1998; Moreno-Hagelsieb and Latimer 2008; Tatusov *et al.* 1997) using tBLASTx with default settings (Camacho *et al.* 2009) and version 6.05 of the *D. melanogaster* genome (Drosophila 12 Genomes Consortium 2007; McQuilton *et al.* 2012). The median *e*-value of each reciprocal comparison was zero, indicating that most orthologs are very similar to one another. Furthermore, when comparing *D. serrata* genes to *D. melanogaster*, the largest *e*-value was 1.6 with only 85 orthologs having an *e*-value > 1e^−10^. Similarly, when comparing *D. melanogaster* genes to *D. serrata*, the largest *e*-value was 0.18 with only 78 orthologs having an *e*-value > 1e^−10^. The correlation between *e*-values for the reciprocal BLAST was 0.88.

### Conclusions

We have assembled a draft genome for a species with no existing genome using only 3GS data. Our study indicates the feasibility of long-read-only genome assembly for nonmodel species with modest sized genomes when using an inbred line. While either greater 3GS coverage or a hybrid merged assembly (Chakraborty *et al.* 2016) may be required to provide greater genome contiguity, it is clear that the genome has a high degree of completeness in terms of gene content and that misassemblies at chromosome arm-level are rare. The genome and its initial annotation provide a useful resource of future population genomic and trait mapping studies in this species.

## Supplementary Material

Supplemental material is available online at www.g3journal.org/lookup/suppl/doi:10.1534/g3.116.037598/-/DC1.
