# Supplementary figures and images for: Single-Molecule Sequencing of the Drosophila serrata Genome
Source: G3 (Bethesda). 2017 Jan 30;7(3):781–8. doi: 10.1534/g3.116.037598 (PMC5345708; doi:10.1534/g3.116.037598)

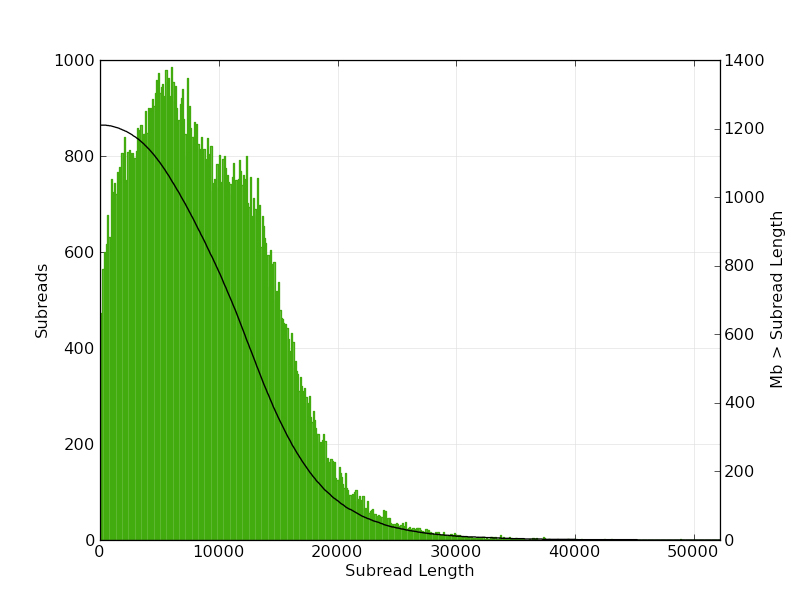

Supplement: Supplementary file 1 [file 781Figure_S1.jpg]
